# Mesenchymal stem cells improve mouse non-heart-beating liver graft survival by inhibiting Kupffer cell apoptosis via TLR4-ERK1/2-Fas/FasL-caspase3 pathway regulation

**DOI:** 10.1186/s13287-016-0416-y

**Published:** 2016-10-27

**Authors:** Yang Tian, Jingcheng Wang, Wei Wang, Yuan Ding, Zhongquan Sun, Qiyi Zhang, Yan Wang, Haiyang Xie, Sheng Yan, Shusen Zheng

**Affiliations:** 1 Key Laboratory of Combined Multi-organ Transplantation, Ministry of Public Health, Key Laboratory of Organ Transplantation, Hangzhou, Zhejiang Province China; 2 Division of Hepatobiliary and Pancreatic Surgery, Department of Surgery, the First Affiliated Hospital, School of Medicine, Zhejiang University, Hangzhou, China; 3 Collaborative Innovation Center for Diagnosis Treatment of Infectious Diseases, Hangzhou, Zhejiang Province China

**Keywords:** Mesenchymal stem cells, Donation after cardiac death, Graft survival, Kupffer cells, Apoptosis

## Abstract

**Background:**

Liver transplantation is the optimal treatment option for end-stage liver disease, but organ shortages dramatically restrict its application. Donation after cardiac death (DCD) is an alternative approach that may expand the donor pool, but it faces challenges such as graft dysfunction, early graft loss, and cholangiopathy. Moreover, DCD liver grafts are no longer eligible for transplantation after their warm ischaemic time exceeds 30 min. Mesenchymal stem cells (MSCs) have been proposed as a promising therapy for treatment of certain liver diseases, but the role of MSCs in DCD liver graft function remains elusive.

**Methods:**

In this study, we established an arterialized mouse non-heart-beating (NHB) liver transplantation model, and compared survival rates, cytokine and chemokine expression, histology, and the results of in vitro co-culture experiments in animals with or without MSC infusion.

**Results:**

MSCs markedly ameliorated NHB liver graft injury and improved survival post-transplantation. Additionally, MSCs suppressed Kupffer cell apoptosis, Th1/Th17 immune responses, chemokine expression, and inflammatory cell infiltration. In vitro, PGE2 secreted by MSCs inhibited Kupffer cell apoptosis via TLR4-ERK1/2-caspase3 pathway regulation.

**Conclusion:**

Our study uncovers a protective role for MSCs and elucidates the underlying immunomodulatory mechanism in an NHB liver transplantation model. Our results suggest that MSCs are uniquely positioned for use in future clinical studies owing to their ability to protect DCD liver grafts, particularly in patients for whom DCD organs are not an option according to current criteria.

**Electronic supplementary material:**

The online version of this article (doi:10.1186/s13287-016-0416-y) contains supplementary material, which is available to authorized users.

## Background

Liver transplantation was first introduced by Dr. Starzl over five decades ago, and is still considered to be the optimal treatment for end-stage liver disease [1]. This procedure is currently accepted and performed by many transplant centres worldwide, but organ shortages remain a prevalent problem [2] which is reflected in the median time to transplant among waiting-listed candidates. For example, in the United States, the median time increased from 14.8 months in 2004 to 19.5 months in 2011 [3]. As a result, death rates among patients on the waiting list have increased over the past two decades [2]. Therefore, donation after cardiac death (DCD), which would expand the pool of available donors, has been proposed as an alternative to ameliorate the organ shortage crisis. In the United States, the percentage of DCD organs has grown annually from 1 % in 1996 to 10 % in 2007 [4]. However, liver graft ischaemia/reperfusion injury (IRI) during DCD donation is inevitable, and the incidence of primary graft dysfunction, early graft loss, and cholangiopathy is fairly high [5–9]. Evidence-based medicine has demonstrated the relatively good quality and safety of DCD grafts with a warm ischaemic time of less than 30 min, and this criterion is implemented in transplantation centres worldwide. For grafts with warm ischaemic time less than 30 min, the primary negative outcome of DCD liver transplantation is late ischaemic cholangiopathy rather than primary non-function, whereas for grafts with warm ischaemic time over 30 min, the primary negative outcome of DCD liver transplantation is early mortality. Numerous studies have been carried out in recent years to identify novel strategies to alleviate DCD liver graft injury.

Mesenchymal stem cells (MSCs) are multipotent cells that are capable of differentiating into specific osteoblastic, adipogenic, and chondrogenic lineages [10]. With the development of regenerative and translational medicine, MSCs have become a subject of research interest because of their low immunogenicity and immunomodulatory properties [11]. MSCs exert powerful immunosuppressive functions via paracrine effects and cell–cell contact-dependent interactions, and MSCs also inhibit the proliferation of activated lymphocytes, thereby providing survival signals to resting immune cells and subsequently stimulating the induction of regulatory immune cells [12]. Among various soluble mediators, inducible nitric oxide synthase (iNOS), transforming growth factor (TGF)β, indoleamine 2,3-dioxygenase (IDO), and prostaglandin E2 (PGE2) are the primary factors responsible for the therapeutic potential of MSCs [13]. Recently, MSCs have been applied to treat autoimmune diseases, diabetes, liver diseases such as fibrosis, non-alcoholic fatty liver cirrhosis, fulminant hepatic failure, and solid organ transplantation [14–18]. However, it is unclear whether MSCs effectively protect DCD liver grafts, and the mechanisms underlying this hypothetical protection remain unknown.

The mouse orthotopic liver transplantation model has unique advantages over rat or swine models, owing to the well-characterized mouse genome and the widespread availability of genetic modification techniques, thus making it a powerful research tool to investigate liver biology, transplant immunology, and DCD-related IRI [19]. However, no optimal mouse non-heart-beating (NHB) liver transplantation model for DCD research has been reported. In this study, we established a standard arterialized mouse NHB liver transplantation model and explored the optimal conditions for NHB modelling. Using this model, we provide the first demonstration of the ability of MSCs to effectively ameliorate NHB-induced graft injury and significantly improve recipient survival by inhibiting Kupffer cell apoptosis. In addition, this study highlights the importance of Kupffer cells in the protective effects exerted by MSCs on NHB liver grafts. Furthermore, MSC-mediated protection against Kupffer cell apoptosis is primarily facilitated by secreted PGE2 via TLR4-ERK1/2-Fas/FasL-caspase3 signalling pathway regulation.

## Methods

### Mice

Male C57Bl/6 (H_2_^b^) and CBF1 (H_2_^bd^) mice aged 8–12 weeks were purchased from the Model Animal Research Center of Nanjing University. All mice were maintained in autocephalous individually ventilated cages (IVC) in a specific-pathogen-free environment and given standard laboratory chow and tap water ad libitum. Animal feeding practices and all experiments involving animals were conducted in accordance with the Guidelines for the Care and Use of Laboratory Animals and received the approval of the Animal Ethics Committees of Zhejiang University (Hangzhou, China).

### Establishment of an arterialized mouse NHB liver transplantation model

C57Bl/6 mice were used as donors, and CBF1 mice were used as recipients. Mouse NHB orthotropic liver transplantation was performed by two licensed animal surgeons using the “double cuff” method, as described in previous studies [20, 21], and the hepatic artery was reconstructed via an aortic trunk method [22]. Briefly, each donor was injected with 100 IU heparin via the tail vein under inhalation anaesthesia. Then, a cross incision was made to expose the donor liver, and the ligaments around the liver were dissected. CO_2_ asphyxiation was administered. NHB timing began when cardiac arrest was confirmed by palpation. After NHB time at 0 min, 5 min, 10 min and 20 min, liver grafts were perfused with cold University of Wisconsin perfusate, harvested with the hepatic–celiac axis–aortic artery segment, and preserved in UW solution at 4 °C. The suprahepatic inferior vena cava was connected using a continuous suture approach, and the portal vein and the infrahepatic inferior vena cava were reconstructed via the “double cuff” method. The common bile duct was connected with a biliary stent. Subsequently, portal vein commencement was carried out at a cold ischaemic time of 120 min. To perform artery reconstruction, the hepatic–celiac axis–aortic artery segment was anastomosed to the recipient aorta by using an “end-to-side anastomosis” technique involving interrupted sutures. Because surgical fault may lead to uncontrolled arterial bleeding or thrombus and result in recipient death, only recipients with perfect hepatic arteriopalmus and without unexpected bleeding were included into our experiments to avoid any surgical influence on recipient survival. After transplantation, all recipients received subcutaneous injections of saline and antibiotics and were maintained in a temperature-controlled IVC unit with a 12 h light–dark cycle and given free access to standard laboratory chow and tap water. Recipient survival was observed and recorded every 6 h.

### Isolation, culture, and retroviral transduction of immortalized T40-MSCs

Immortalized T40-MSCs originally isolated from the bone marrow of C57Bl/6 mice were a kind gift from Prof. Weimin Fan of Zhejiang University. In brief, a male 8-week-old C57Bl/6 mouse was sacrificed, and whole bone marrow was retrieved by flushing the bones with Dulbecco’s modified Eagle’s medium (DMEM; GIBCO, Waltham, MA, USA). All bone marrow cells were cultured in DMEM with 10 % foetal bovine serum (FBS; GIBCO) for 48 h. Non-adherent haematopoietic cells were discarded. Adherent cells were then washed three times with phosphate-buffered saline (PBS) and replenished with 15 mL fresh DMEM containing 10 % FBS. When the cells grew to 60 % confluence, they were transfected with a T40-expressing retroviral vector.

To achieve T40-expressing retrovirus packaging, HEK293 cells were plated at a cell density of approximately 50 %. After 3–5 h, the cells were transfected with 250 μL Opti-MEM plus 37.5 μL of a solution containing 15 μL Lipofect AMINE (Invitrogen, Waltham, MA, USA), 7.5 μL of the packaging plasmid pAmpho and 15 μL pSSRV60-T40. The medium was discarded 3–5 h later and replaced with 4 mL fresh complete DMEM. Retrovirus-containing supernatants were collected at 36, 60, and 84 h. The generated virus was kept at 4 °C or directly used to infect MSCs. Immortalized T40–MSCs were generated by transducing MSCs twice per day for 2 consecutive days. Hygromycin B (0.2 mg/mL; Sigma-Aldrich, St. Louis, MO, USA) selection began at the end of the second round of infection.

### Differentiation and identification of MSCs

Adipogenic and osteogenic differentiation were induced in vitro, and MSC identification was carried out. To achieve adipogenic differentiation, MSCs were cultured with 1 μM dexamethasone and 0.2 mM indomethacin in DMEM plus 10 % FBS, and the conditional medium was changed every other day. After 7 days, adipocytic differentiation was examined through oil red O staining. Osteogenic MSC differentiation was performed with conditional medium containing 100 mM dexamethasone, 10 mM β-glycerophosphate disodium, and 50 μM l-ascorbic acid-2-phosphate. The medium was changed every other day, and 2 weeks later MSCs were stained with alizarin red to examine calcium deposition. All conditional medium supplements were purchased from Sigma-Aldrich.

### MSC infusion and GdCl_3_ administration

MSCs in the logarithmic phase were trypsinised and suspended in DMEM at a concentration of 5 × 10^7^/mL. After portal vein commencement, a 20-μL suspension containing 10^6^ MSCs was injected intrasplenically. Electrocoagulation was applied at the puncture point to stop bleeding. In other experiments, 20 mg/Kg gadolinium chloride (GdCl_3_; Sigma-Aldrich) solution was administered intraperitoneally to donor C57Bl/6 mice 24 h before transplantation to eliminate intrahepatic Kupffer cells.

### Liver function test and histological evaluation

Under inhalation anaesthesia, blood was collected from the inferior vena cava, and serum alanine aminotransferase (ALT) and aspartate aminotransferase (AST) were measured using an automatic biochemical analyser in the clinical laboratory of our hospital. Liver grafts were harvested, formalin-fixed, sectioned, and stained with haematoxylin and eosin for histopathological evaluation using the Suzuki Score System (Table 1). Each graft section was examined in a blinded fashion by an experienced pathologist.

**Table 1:** Standards of Suzuki score for liver injury

Score	Congestion	Vacuole degeneration	Necrosis
0	None	None	None
1	Slight	Slight	Single cell
2	Mild	Mild	<30 %
3	Moderate	Moderate	31–60 %
4	Severe	Severe	>60 %

### Quantitative real-time polymerase chain reaction

The mRNA levels of cytokines and chemokines were detected by quantitative real-time polymerase chain reaction (RT-PCR), as described in our previous study [20, 23]. Briefly, total RNA was extracted from frozen grafts by using TRIzol reagent (Invitrogen) and then reverse-transcribed into cDNA using a reverse transcriptase kit (Bio-Rad, Hercules, CA, USA). cDNA primers and iTaq Universal SYBR Green Supermix (Bio-Rad) were added to PCR plates, and PCR was carried out with an Applied Biosystems 7900 PCR System. Relative mRNA quantification was performed using the 2^−△△Ct^ method according to the manufacturer’s manual. Gene-specific primers are shown in Table 2.

**Table 2:** Sequences of primers used in quantitative RT-PCR

Gene	Sequences		Length (base)
β-actin	Forward:	CATTGCTGACAGGATGCAGAAGG	23
	Reverse:	TGCTGGAAGGTGGACAGTGAGG	22
IL1α	Forward:	ACGGCTGAGTTTCAGTGAGACC	22
	Reverse:	CACTCTGGTAGGTGTAAGGTGC	22
IL1β	Forward:	TGGACCTTCCAGGATGAGGACA	22
	Reverse:	GTTCATCTCGGAGCCTGTAGTG	22
IL2	Forward:	GCGGCATGTTCTGGATTTGACTC	23
	Reverse:	CCACCACAGTTGCTGACTCATC	22
IL4	Forward:	ATCATCGGCATTTTGAACGAGGTC	24
	Reverse:	ACCTTGGAAGCCCTACAGACGA	22
IL6	Forward:	TACCACTTCACAAGTCGGAGGC	22
	Reverse:	CTGCAAGTGCATCATCGTTGTTC	23
IL10	Forward:	CGGGAAGACAATAACTGCACCC	22
	Reverse:	CGGTTAGCAGTATGTTGTCCAGC	23
TNFα	Forward:	GGTGCCTATGTCTCAGCCTCTT	22
	Reverse:	GCCATAGAACTGATGAGAGGGAG	23
IFNγ	Forward:	CAGCAACAGCAAGGCGAAAAAGG	23
	Reverse:	CAGCAACAGCAAGGCGAAAAAGG	23
F4/80	Forward:	CGTGTTGTTGGTGGCACTGTGA	22
	Reverse:	CCACATCAGTGTTCCAGGAGAC	22

*IFN* interferon, *IL* interleukin, *RT-PCR* real-time polymerase chain reaction, *TNF* tumour necrosis factor

### Immunofluorescence and terminal deoxynucleotidyl transferase dUTP nick end labelling (TUNEL) detection

For immunofluorescence analysis, frozen sections of liver graft specimens were incubated with anti-mouse F4/80-FITC (Abcam, Cambridge, MA, USA) at 4 °C overnight.

Proliferating Kupffer cells in frozen graft sections were stained with anti-mouse F4/80-Alexa Flour 647 (Abcam) and anti-Ki67 antibodies (Abcam) at 4 °C overnight. Sections were then incubated with a goat-anti-rat IgG-Alexa Flour 488 secondary antibody (Abcam) for 2 h at room temperature.

Apoptotic Kupffer cells in frozen graft sections were stained with anti-mouse F4/80-Alexa Flour 647 (Abcam) and detected in a TUNEL assay with an ApopTag® Plus Peroxidase in Situ Apoptosis Kit (EMD Millipore, Darmstadt, Germany). Detection and all measurements were carried out following the manufacturer’s instructions.

All of the sections were then mounted in Mounting Medium with DAPI (Vector Laboratories, Burlingame, CA, USA) and scanned with a two-photon laser confocal microscope (Olympus, Tokyo, Japan).

### Immunohistochemistry staining

For immunohistochemistry staining, antigen retrieval and goat serum blocking were first performed, and then paraffin-embedded graft sections were incubated with anti-myeloperoxidase (MPO; Abcam) and anti-CD3 (R&D, Minneapolis, MN, USA) at 4 °C overnight. Sections were then incubated with a goat-anti-rabbit or goat-anti-rat IgG secondary antibody conjugated to horseradish peroxidase for 2 h at room temperature (Invitrogen). 3,3′-diaminobenzidine peroxidase substrate was used as a detection reagent (Vector Laboratories). All sections were visualized using BX41 microscopy (Olympus).

### Immunoassay and MPO measurement

Serum cytokine and chemokine protein levels were measured with a Luminex-200 system using the ProcartaPlex Mouse Cytokine & Chemokine Panel Kit (eBioscience, San Diego, CA, USA), and serum MPO activity was measured using an MPO Activity Colorimetric Assay Kit (Biovision, Milpitas, CA, USA). Detection and all measurements were carried out following the manufacturer’s instructions.

### MSC–macrophage in vitro co-culture

In vitro experiments were carried out using the mouse-derived RAW264.7 cell line, which was purchased from the Chinese Academy of Sciences, to represent Kupffer cells. Generally, 5 × 10^5^ macrophages per well were seeded in a six-well plate, and 10^5^ MSCs were seeded in a 0.4-μm polycarbonate membrane insert Transwell (Corning, Corning, NY, USA) in medium containing or lacking NS-398 (5 μM; Sigma-Aldrich). After 24 h, the medium was changed once to remove non-adherent dead cells. After 2 h of incubation at 37 °C without O_2_, the macrophages were transferred into co-culture with the Transwells containing MSCs, and 0, 100, 200, 400, or 800 μM hydrogen peroxide (H_2_O_2_) was added to specific wells. Then, the co-cultured cells were incubated at 37 °C with 5 % CO_2_ for 6 h to mimic the process of IRI during NHB liver transplantation in vivo.

### Flow cytometry to detect co-cultured macrophage apoptosis

After 6 h of co-culture, all macrophages were collected for early apoptosis detection by flow cytometry using an Annexin V-FITC/PI apoptosis detection kit (DOJINDO, Kumamoto, Japan) according to the manufacturer’s manual.

### Western blotting

The expression of specific target proteins was examined by Western blotting, as described in our previous study [23]. Total protein fractions from co-cultured macrophages were extracted with RIPA and protease and phosphatase inhibitor cocktail (Thermo Fisher, Waltham, MA, USA). Anti-TLR2 (Epitomics, Cambridge, MA, USA), anti-TLR4 (Abcam), anti-ERK1/2 (Cell Signaling Technology, Danvers, MA, USA), anti-pERK1/2 (Cell Signaling Technology), anti-Fas (Biovision, Milpitas, CA, USA), anti-FasL (Biovision), anti-cleaved caspase3 (Cell Signaling Technology), and anti-β-actin (Abcam) antibodies were incubated with macrophage protein-containing nitrocellulose membranes overnight. A ChemiDoc Imaging System was used to develop the images (Bio-Rad, Hercules, CA, USA). Grey values were calculated and analysed for each band with ImageLab software (Bio-Rad).

### Statistics

Recipient survival was expressed graphically using the Kaplan–Meier method. All other data are presented as the mean ± SEM and were analysed using GraphPad Prism 5.0.1 software (GraphPad Prism Software Inc., La Jolla, CA, USA). A log-rank (Mantel-Cox) test was used for survival rate analysis, and Student’s *t* test was applied to assess data obtained from different groups. Two-sided *P* < 0.05 was considered to be statistically significant.

## Results

### NHB time extension reduces the survival rate with aggravated liver graft injury

**Fig. 1:**
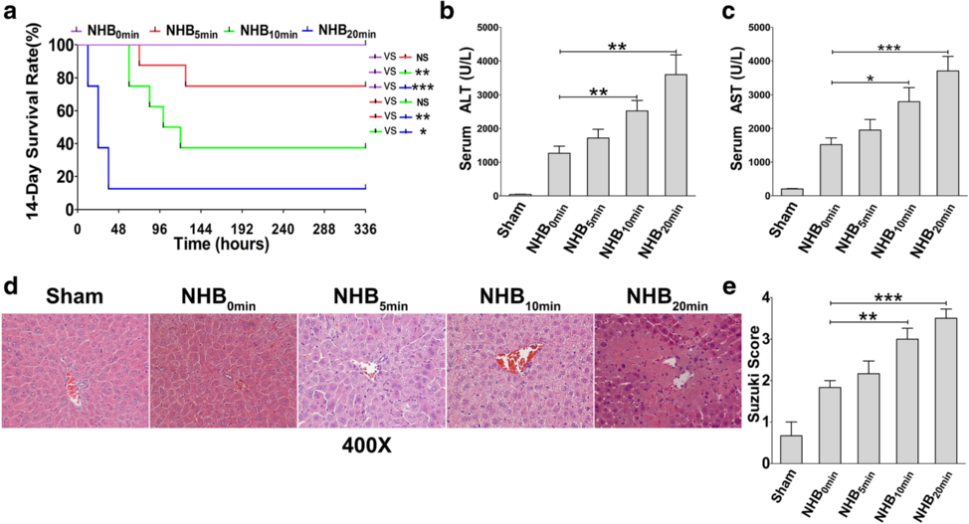
Non-heart-beating (*NHB*) time extension reduces the survival rate with aggravated liver graft injury. Arterialized C57Bl/6-CBF1 liver transplantations were performed with 0 min, 5 min, 10 min, and 20 min of NHB time, and 120 min of cold ischaemic time, respectively. **a** Cumulative recipient survival rates were analysed. Results of four independent experiments were combined (*N* = 8 for each group). Log-Rank (Mantel-Cox) test was used for comparison of different survival curves. *P < 0.05, **P < 0.01, ***P < 0.001. Serum **b** alanine aminotransferase (*ALT*) and **c** aspartate aminotransferase (*AST*) levels from each group were tested at 6 h post-transplantation (*N* = 6 for each group). **d** Liver grafts were sectioned for histological examination. Representative images from one experiment are shown at 400× magnification. **e** Suzuki scores of each section were evaluated by an experienced pathologist in a blinded fashion (*N* = 6 for each group). **b**, **c**, **e** Results are presented as mean ± SEM for each group. *P < 0.05, **P < 0.01, ***P < 0.001. *NS* not significant

To establish an optimal mouse model for NHB liver graft research, arterialized orthotopic liver transplantations were performed, and different NHB timing options were tested. Sham operations were carried out as normal controls. For the NHB_0min_ group, all recipients survived more than 14 days post-transplantation. Only 75 % of recipients survived at 14 days in the NHB_5min_ group, and the 14-day survival rate was reduced to 37.5 % and 12.5 % in the NHB_10min_ group and NHB_20min_ group, respectively, thus revealing the increased incidence of primary graft dysfunction and early graft dysfunction with NHB time extension (Fig. 1a). Serum levels of ALT/AST, the most sensitive IRI indicator in liver grafts, rise rapidly once reperfusion begins and reach a peak at 6 h post-transplantation. Afterwards, the liver graft initiates certain mechanisms to repair IRI and hepatocellular damage, and serum ALT/AST levels decrease gradually. However, if IRI is too severe for the graft to repair itself, liver dysfunction occurs within several days, ultimately resulting in recipient death [24–26]. To evaluate liver graft injury, serum and liver specimens were collected and examined at 6 h post-transplantation when inflammation peaked. A gradual increase in serum ALT and AST levels was detected in these groups (Fig. 1b and c). Histopathological examination indicated 30 % hepatocellular oedema with mild inflammatory cell infiltration in the NHB_0min_ group, 60 % hepatocellular oedema with widened hepatic sinusoids in the NHB_5min_ group, severe hepatocellular oedema with spotty necrosis in the NHB_10min_ group, and patchy necrosis accompanied by severe inflammatory cell infiltration and widened sinusoids in the NHB_20min_ group (Fig. 1d). These results were consistent with a clear increase in the Suzuki scores of the liver grafts (Fig. 1e). Together, these data suggested that NHB time extension aggravated liver graft injury, resulting in a dramatic reduction in the survival rate after liver transplantation.

### Impaired Th1/Th2 responses and Kupffer cells after NHB liver transplantation

**Fig. 2:**
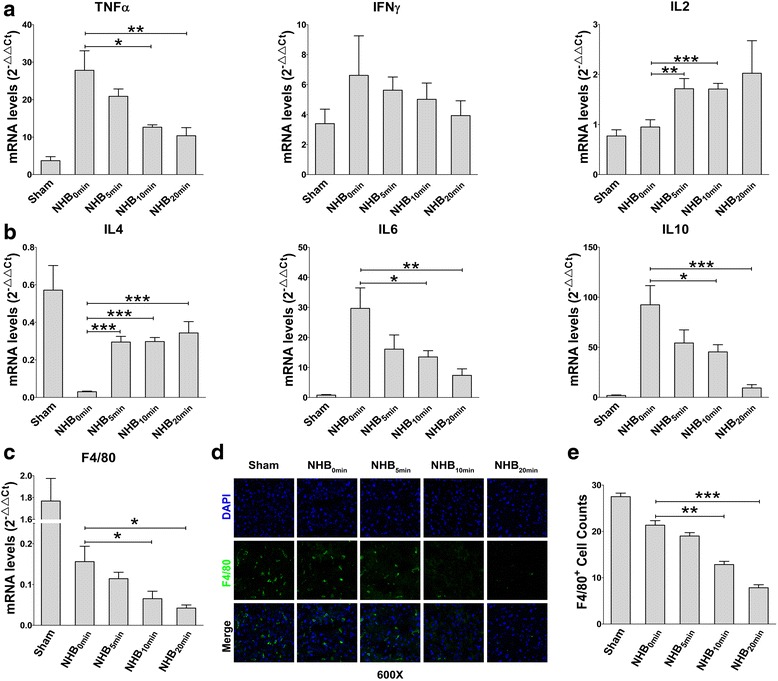
Th1/Th2 responses and Kupffer cells are impaired after non-heart-beating (*NHB*) liver transplantation. At 6 h post-transplantation, liver grafts from each group were collected for quantitative RT-PCR analysis and immunofluorescence staining. Intrahepatic mRNA levels of **a** Th1 cytokines (TNFα, IFNγ, and IL2) and **b** Th2 cytokines (IL4, IL6, and IL10) in each group were measured by quantitative RT-PCR (*N* = 8–10 for each group). **c** Intrahepatic mRNA level of F4/80 was detected by quantitative RT-PCR (*N* = 8–10 for each group). **d** F4/80 immunofluorescence staining of frozen liver graft tissues were visualized by confocal scanning. Representative images from one experiment are shown at 600× magnification. **e** Intrahepatic F4/80-positive cells in each sections were counted for statistical analysis (*N* = 6 for each group). **a**, **b**, **c**, **e** Results are presented as mean ± SEM for each group. *P < 0.05, **P < 0.01, ***P < 0.001. *IFN* interferon, *IL* interleukin, *TNF* tumour necrosis factor

Th1 (tumour necrosis factor (TNF)α, interferon (IFN)γ, and interleukin (IL)2) and Th17 (IL17) cytokines are pro-inflammatory during pathogenesis, whereas Th2 cytokines (IL4, IL6, and IL10) exert immunomodulatory functions that inhibit severe inflammation in the body. Given the critical role of Th1/Th2 immune responses in liver self-homeostasis during pathological events [27], the intrahepatic mRNA expression of classical Th1/Th2 cytokines was determined by performing quantitative RT-PCR. Unexpectedly, Th1 and Th2 cytokine mRNA levels did not increase with aggravated liver graft injury when NHB time was extended. In contrast, with the exception of IL2 and IL4, the mRNA expression of all other Th1 and Th2 cytokines, including TNFα, IFNγ, IL6, and IL10, was downregulated, thus indicating the impairment of intrahepatic Th1/Th2 cell responses after NHB liver transplantation (Fig. 2a and b). Kupffer cells are very sensitive to pathological factors during IRI, and initiate Th1/Th2 responses by secreting certain cytokines and chemokines. To investigate Kupffer cell status in the context of Th1/Th2 response impairment, we first detected the intrahepatic mRNA expression of F4/80, a classic surface marker of Kupffer cells [28]. As expected, F4/80 mRNA levels decreased robustly as NHB time was extended (Fig. 2c). Similarly, immunofluorescence staining of frozen grafts showed a dramatic loss of F4/80-positive Kupffer cells within different groups (Fig. 2d and e). Thus, as NHB time was extended, aggravated liver IRI led to a dramatic loss of Kupffer cells and impaired Th1/Th2 immune responses post-transplantation.

In addition, we compared survival rates, ALT and AST levels, and graft histopathological injury in different NHB groups and determined the optimal conditions for the mouse arterialized NHB liver transplantation model to be an NHB time of 10 min and a cold ischaemic time of 120 min; under these conditions, any effects of various interventions were conveniently observed as changes in recipient survival.

### MSCs ameliorate NHB-induced graft injury and increase the recipient survival rate

**Fig. 3:**
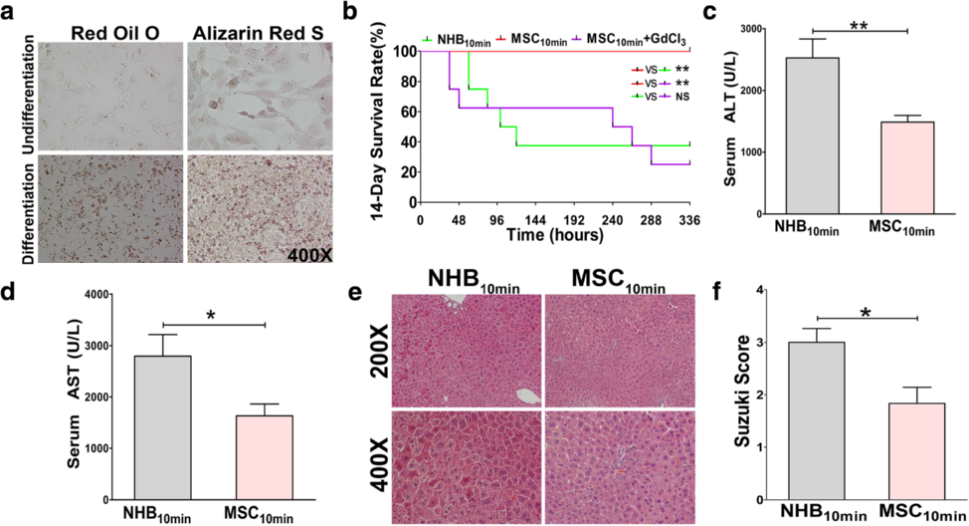
Mesenchymal stem cells (*MSCs*) ameliorate non-heart-beating (*NHB*)-induced graft injury and increase the recipient survival rate. Arterialized C57Bl/6-CBF1 liver transplantations were performed with 10 min of NHB time and 120 min of cold ischaemic time, respectively. In the MSC_10min_ group, 10^6^ MSCs were intrasplenically infused post-transplantation. In the MSC_10min_ + GdCl_3_ group, 20 mg/Kg GdCl_3_ was intraperitoneally injected 24 h before graft harvest. **a** MSCs were stained with red oil O and alizarin red S for adipogenic and osteogenic identification after conditioned differentiation. Undifferentiation: normal culture medium; Differentiation: conditioned culture medium. Representative images from one experiment out of three are shown at 400× magnification. **b** Cumulative recipient survival rates were analysed. Data from three independent experiments are combined (*N* = 8 for each group). Log-Rank (Mantel-Cox) test was used for comparison of different survival curves. *P < 0.05, **P < 0.01. Serum **c** alanine aminotransferase (*ALT*) and **d** aspartate aminotransferase (*AST*) levels from each group were tested at 6 h post-transplantation (*N* = 6 for each group). **e** Liver grafts were sectioned for histological examination. Representative images from one experiment are shown at 200× and 400× magnification. **f** Suzuki scores of each section were evaluated by an experienced pathologist in a blinded fashion (*N* = 6 for each group). **c**, **d**, **f** Results are presented as mean ± SEM for each group. *P < 0.05, **P < 0.01. *NS* not significant

We evaluated the adipogenic and osteogenic differentiation potential of MSCs in conditioned medium in vitro. Intra-MSC lipid droplets stained with oil red O [29] and calcium deposition stained with alizarin red S were clearly observed (Fig. 3a) [30].

To examine the role of MSCs in mouse NHB liver transplantation, MSCs were slowly intrasplenically infused after portal vein commencement; under these conditions, the majority of MSCs gradually migrate into the hepatic sinusoids through the portal vein and avoid portal vein embolization [31–33]. MSC infusion effectively improved the survival rate post-transplantation, with 100 % survival of recipients by day 14, compared with 37.5 % survival in the NHB_10min_ group (Fig. 3b), a result consistent with a significant decrease in serum ALT and AST levels at 6 h post-transplantation (Fig. 3c and d). Histopathological examination showed normal hepatocellular morphology without oedema, vacuolar degeneration, or necrosis, and a significant decrease in the Suzuki scores in the MSC_10min_ group, which significantly differed from the severe hepatocellular oedema with spotty necrosis observed in the NHB_10min_ group (Fig. 3e and f). Thus, MSC infusion significantly ameliorated warm ischaemia-induced liver graft injury and promoted recipient survival after NHB liver transplantation.

### MSCs protect and restore Kupffer cells by inhibiting apoptosis but not by promoting proliferation

**Fig. 4:**
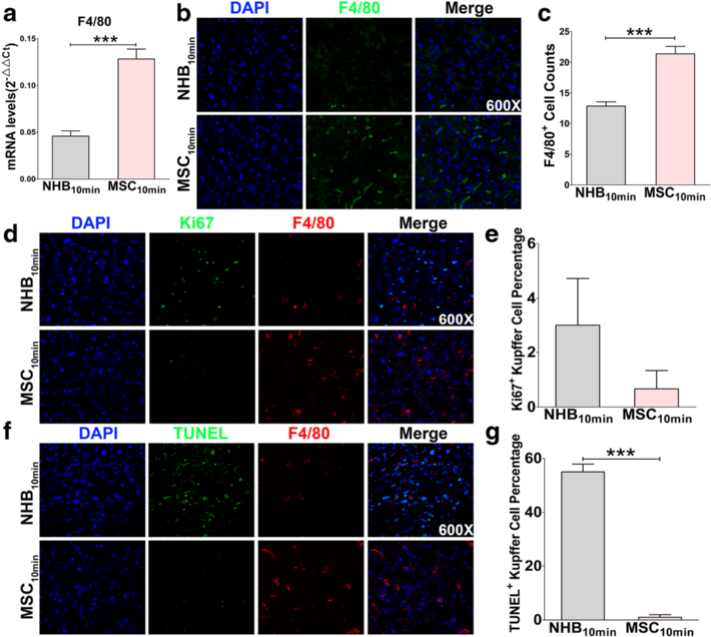
Mesenchymal stem cells (*MSCs*) protect and restore Kupffer cells by inhibiting apoptosis but not by promoting proliferation. At 6 h post-transplantation, liver grafts from each group were collected for quantitative RT-PCR analysis and immunofluorescence staining. **a** Intrahepatic mRNA level of F4/80 was detected by quantitative RT-PCR (*N* = 6 for each group). **b** F4/80 immunofluorescence staining of frozen liver graft tissues were visualized by confocal scanning. Representative images from one experiment are shown at 600× magnification. **c** Intrahepatic F4/80-positive cells in each section were counted for statistical analysis (*N* = 6 for each group). **d** Proliferation of Kupffer cells was detected by Ki67 (*green*) and F4/80 (*red*) double staining. Representative images from one experiment are shown at 600× magnification. **e** The percentage of Ki67^+^ F4/80^+^ cells in total F4/80^+^ Kupffer cells was counted and calculated for statistical analysis (*N* = 3 for each group). **f** Apoptosis of Kupffer cells was detected by TUNEL (*green*) and F4/80 (*red*) double staining. Representative images from one experiment are shown at 600× magnification. **g** The percentage of TUNEL^+^ F4/80^+^ cells in total F4/80^+^ Kupffer cells was counted and calculated for statistical analysis (*N* = 3 for each group). **a**, **c**, **e**, **g** Results are presented as mean ± SEM for each group. ***P < 0.001. *NHB* non-heart-beating, *TUNEL* terminal deoxynucleotidyl transferase dUTP nick end labelling

As mentioned above, Kupffer cells were dramatically impaired by extended NHB injury, thus indicating a close relationship between graft quality and Kupffer cell status. Because MSCs effectively protect against NHB liver graft injury, we sought to determine how MSC infusion affects Kupffer cells. To investigate this question, F4/80 mRNA expression and immunofluorescence staining were evaluated. Interestingly, MSCs significantly improved F4/80 mRNA expression in liver grafts compared with the NHB_10min_ group (Fig. 4a). Moreover, an obvious increase in F4/80-positive cells was detected using confocal microscopy (Fig. 4b and c), thus suggesting the restoration of Kupffer cells due to MSC infusion.

However, it is unclear how MSCs promote Kupffer cell restoration. Thus, in situ proliferating and apoptotic Kupffer cells were detected by performing Ki67-F4/80 and TUNEL-F4/80 immunofluorescent staining, respectively. Proliferating cells were mainly non-parenchymal cells in the NHB_10min_ group, while proliferating cells could seldom be detected in the MSC_10min_ group (Fig. 4d). However, the percentage of Ki67-positive Kupffer cells showed no difference between these two groups (Fig. 4e). Meanwhile, sporadic hepatocellular apoptosis and non-parenchymal cell apoptosis were detected in the NHB_10min_ group, which was rarely observed in the MSC_10min_ group (Fig. 4f). Besides, the percentage of TUNEL-positive Kupffer cells in the NHB_10min_ group was significantly higher than in the MSC_10min_ group (Fig. 4g). These results indicated that MSCs protected Kupffer cells by inhibiting their apoptosis but not by promoting their proliferation. We hypothesized that the initiation of certain proliferative mechanisms by non-parenchymal cells might replace specific cells lost due to apoptosis resulting from severe injury and graft damage in the NHB_10min_ group. While MSC infusion reversed the damage caused by IRI, non-parenchymal cells and hepatocytes rarely showed Ki67 and TUNEL expression. However, the underlying mechanisms require further investigation.

### MSC-mediated protection of NHB grafts depends on Kupffer cells

Although we observed a close relationship between MSCs and Kupffer cells, we lacked more direct evidence. Because MSCs recruit peripheral lymphocytes into the liver graft by secreting a series of chemokines, further study is needed to determine whether the restoration of F4/80^+^ cells after MSC infusion is related to peripheral macrophage recruitment. The administration of 20 mg/Kg doses of GdCl_3_ causes Kupffer cell apoptosis and selectively blocks Kupffer cell effector functions but does not cause liver parenchymal cell toxicity [31]. Rather than shortening the survival of injected mice, the administration of 20 mg/Kg doses of GdCl_3_ may protect the liver against IRI [34]. To elucidate the true role of Kupffer cells in the MSC-mediated protection of NHB grafts, 20 mg/Kg GdCl_3_ was applied in NHB donors 24 h before surgical procedures, so as not to affect the recipient’s bone marrow-derived macrophage recruitment into the liver graft post-transplantation. After the depletion of intrahepatic Kupffer cells with GdCl_3_, NHB liver grafts were transplanted into recipients with simultaneous MSC infusion. Unexpectedly, the 14-day survival rate dramatically decreased to 25 % in the GdCl_3_-pretreated group (Fig. 3b), and this result was not significantly different from that observed for the NHB_10min_ group. Thus, the protective effects of MSCs on NHB liver grafts appear to depend on Kupffer cells. Furthermore, the restoration of F4/80^+^ cells after MSC infusion is dependent on the direct protection of Kupffer cells against apoptosis, not the recruitment of peripheral macrophages into the liver grafts.

### MSCs suppress Th1/Th17 cells but promote IL10 secretion

**Fig. 5:**
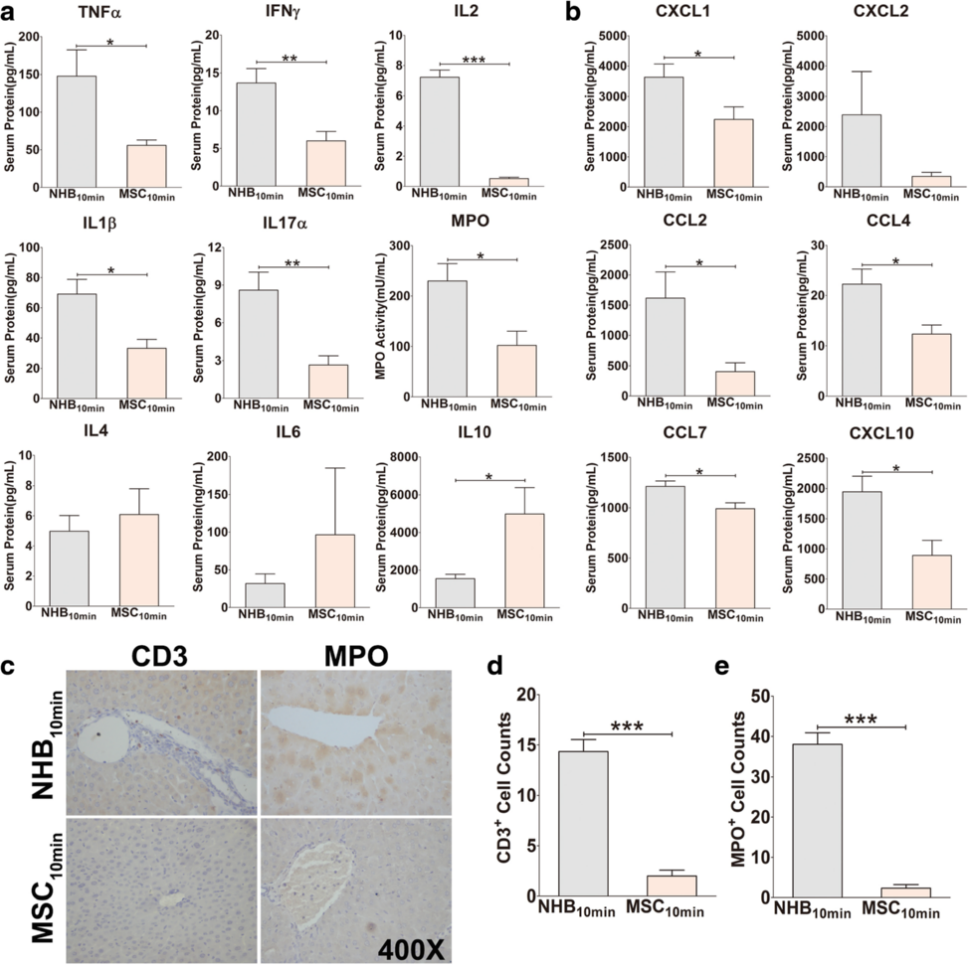
Mesenchymal stem cell (*MSC*) infusion suppresses serum Th1/Th17 cytokines and chemokines and inhibits intrahepatic inflammatory cell infiltration. **a** Serum protein levels of Th1 cytokines (TNFα, IFNγ, and IL2), Th2 cytokines (IL4, IL6, and IL10), Th17 cytokine (IL17), and other pro-inflammatory cytokines (IL1β and MPO) were measured by immunoassay method (*N* = 4–6 for each group). **b** Serum protein levels of CXCL1, CXCL2, CCL2, CCL4, CCL7, and CXCL10 were detected by immunoassay method (*N* = 4–6 for each group). **c** Intrahepatic neutrophil and T-cell infiltration was detected by MPO and CD3 immunohistochemistry staining. Representative images from one experiment are shown at 400× magnification. **d** MPO- and **e** CD3-positive cells were counted for statistical analysis (*N* = 3 for each group). **a**, **b**, **d**, **e** Results are presented as mean ± SEM for each group. *P < 0.05, **P < 0.01, ***P < 0.001. *CCL* C-C motif ligand, *CXCL* C-X-C motif ligand, *IFN* interferon, *IL* interleukin, *MPO* myeloperoxidase, *NHB* non-heart-beating, *TNF* tumour necrosis factor

As mentioned previously, classical cytokines were investigated to assess Th1/Th2/Th17 responses post-transplantation. Compared with the NHB_10min_ group, MSCs together with restored Kupffer cells significantly decreased serum TNFα, IFNγ, and IL2 protein secretion. Similarly, the serum protein expression of other pro-inflammatory cytokines, including IL17, IL1β, and MPO, was suppressed. MSCs had no effects on Th2 cytokines such as IL4 and IL6 but clearly promoted IL10 secretion (Fig. 5a). Thus, MSC infusion suppresses Th1/Th17 immune responses but exerts no effects on Th2 responses with the exception of IL10 secretion.

### MSCs suppress chemokine expression and inflammatory cell infiltration in liver grafts

Kupffer cells are known as “sensor cells” that respond to IRI and release different chemokines, which recruit large amounts of inflammatory cells that infiltrate the liver [35]. The serum protein levels of chemokines were investigated to evaluate changes resulting from MSC infusion. Interestingly, the serum protein expression of CXCL1, CCL2, CCL4, CCL7, and CXCL10 was notably suppressed in the MSC infusion group (Fig. 5b). Intrahepatic infiltrating cells are indicative of the degree of liver inflammation; specifically, neutrophils and T cells are the most informative. Because neutrophils migrate into pathological sites and release MPO, MPO is regarded as a typical neutrophil biomarker, whereas CD3 is a classical T-cell biomarker. We performed immunohistochemical staining to detect MPO and CD3 to evaluate inflammation in liver grafts. Intrahepatic infiltration of MPO^+^ neutrophils and CD3^+^ T cells was dramatically decreased (Fig. 5c, d and e). Our results indicated that Kupffer cell restoration, together with infused MSCs, suppresses chemokine expression and reduces neutrophil and T-cell infiltration into liver grafts post-transplantation.

### MSCs inhibit Kupffer cell apoptosis by secreting PGE2 in vitro

**Fig. 6:**
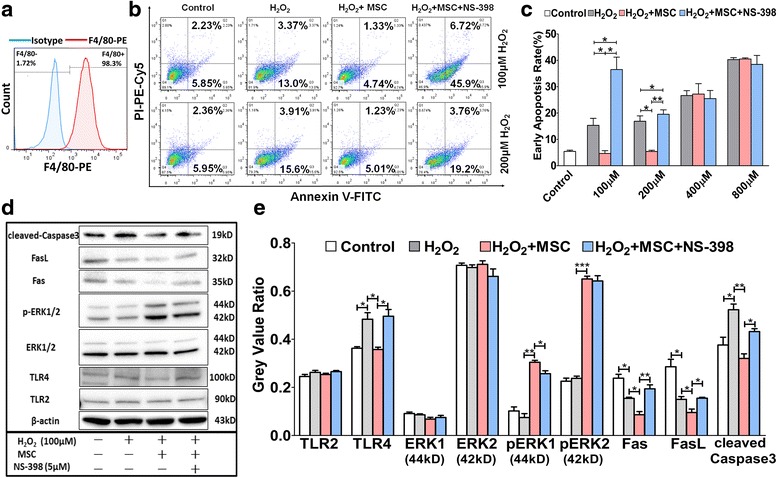
Mesenchymal stem cells (*MSCs*) inhibit Kupffer cell apoptosis via the PGE2-TLR4-ERK1/2-Fas/FasL-caspase3 pathway. Murine-derived RAW264.7 cells were used to represent Kupffer cells; 5 × 10^5^ macrophages per well were seeded in a six-well plate, and 10^5^ MSCs were seeded in a 0.4-μm polycarbonate membrane insert Transwell in medium containing or lacking NS-398 (5 μM). After 24 h, the medium was changed once to remove non-adherent dead cells. After 2 h of incubation at 37 °C without O_2_, the macrophages were transferred into co-culture with the Transwells containing MSCs, and 0, 100, 200, 400, or 800 μM hydrogen peroxide (*H*_*2*_ *O*_*2*_) was added to specific wells. Then, the co-cultured cells were incubated at 37 °C with 5 % CO_2_ for 6 h to mimic the process of IRI during NHB liver transplantation in vivo. **a** RAW264.7 cells were stained with F4/80 antibody, a surface marker of Kupffer cells, and analysed by flow cytometry. Representative images from one experiment are shown. *Blue* shading and *red* shading indicate immunofluorescence intensity of cells for the isotype and F4/80 antibodies, respectively. **b** In vitro co-culture system. After H_2_O_2_ stimulation for 6 hours, apoptosis of Kupffer cells was detected by Annexin V/PI staining. Representative images from one experiment out of three are shown. **c** Summarized results are presented as mean ± SEM (*N* = 3 for each group). **d** After H_2_O_2_ stimulation for 6 hours, expression of TLR2, TLR4, ERK1/2, p-ERK1/2, Fas, FasL, cleaved caspase3, and β-actin from Kupffer cells was determined by Western blotting analysis. Representative images from one experiment are shown. **e** Grey value of each band was calculated by ImageLab software. Representing relative expression of target proteins, the grey value ratio compared with β-actin was analysed and shown as mean ± SEM (*N* = 3 for each group). *P < 0.05, **P < 0.01, ***P < 0.001

To clarify the underlying mechanism by which MSCs inhibit Kupffer cell apoptosis, we used an in vitro cell–cell co-culture system with the murine-derived RAW264.7 cell line to represent Kupffer cells, which exclusively express F4/80 antigen on the membrane surface (Fig. 6a) [36]. Six hours after hydrogen peroxide (H_2_O_2_) stimulation, Kupffer cell apoptosis was elevated in an H_2_O_2_ dose-dependent manner compared with that in controls, whereas co-culture with MSCs protected Kupffer cells from apoptosis in the presence of low concentrations of H_2_O_2_ (100 μM and 200 μM). NS-398, a specific PGE2 inhibitor, dramatically reversed the reduction in apoptosis observed in the presence of MSCs (Fig. 6b and c). When stimulated with high concentrations of H_2_O_2_ (400 μM and 800 μM), MSCs lost their ability to protect against apoptosis, regardless of PGE2 inhibition (Fig. 6c). Thus, MSC-derived PGE2 is a key factor in Kupffer cell apoptosis inhibition. Furthermore, the paracrine protective effects of MSCs on Kupffer cells were restricted, not unlimited, in our in vitro experiments.

### MSC-derived PGE2 plays an essential role in inhibiting Kupffer cell apoptosis by regulating the TLR4-ERK1/2-Fas/FasL-caspase3 signalling pathway

The accumulation of reactive oxidant species elevates TLR4 expression, activates MAPK-induced NFκB translocation, and exacerbates liver injury [37]. Peng et al. have reported the ability of elevated TLR4 to promote PKCζ-induced caspase3 activation of Kupffer cells [38, 39], and in other reports TLR2 and TLR4 have been found to inhibit Bcl-2 expression by reducing AKT and ERK1/2 phosphorylation [40–43]. Because we demonstrated the critical role of PGE2 in the protective effects mediated by MSCs, we performed sequential Western blotting to detect related signalling pathways. PGE2 downregulated TLR4, but not TLR2, expression in Kupffer cells, thus activating ERK1/2 phosphorylation which resulted in the reduced expression of Fas/FasL and cleaved caspase3 (Fig. 6d and e). Thus, PGE2 inhibits Kupffer cell apoptosis by regulating the TLR4-ERK1/2-Fas/FasL-caspase3 signalling pathway.

### Long-term safety of MSC infusion

**Fig. 7:**
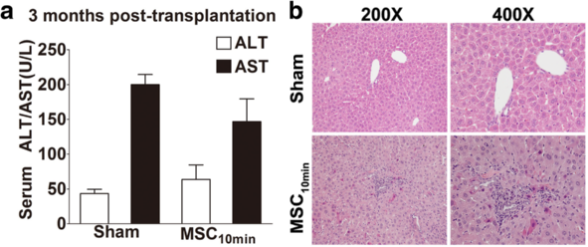
Mesenchymal stem cell (*MSC*) infusion is safe and effective with no tumorigenesis detected during 3 months of observation. **a** At 3 months post-transplantation, serum alanine aminotransferase (*ALT*) and aspartate aminotransferase (*AST*) levels in the MSC_10min_ group showed no difference compared with the sham group. Results are presented as mean ± SEM (*N* = 6 for each group). **b** Liver grafts were harvested for histological examination and no tumorigenesis was detected. Haematoxylin and eosin staining of grafts showed relatively normal hepatocellular morphology, cell infiltration, and mild hyperplasia around the portal area, which may represent a response to ischaemic biliary injury. Representative images are shown at 200× and 400×

The long-term safety of MSC application in vivo is an inevitable question. To evaluate safety in our study, we prolonged our observation period of the MSC infusion group to 3 months. The survival rate at 3 months post-transplantation was 75 %, and all surviving recipients were healthy with normal serum ALT/AST levels (Fig. 7a). No tumourigenesis was detected, although two recipients died because of biliary tract obstruction, as confirmed by autopsy. Histological examination showed a relatively normal hepatocellular morphology, obvious cell infiltration, and mild hyperplasia around the portal area (Fig. 7b), which may represent a response to ischaemic biliary injury. Whether these infiltrated cells were inflammatory cells, such as neutrophils and T cells, or infused MSCs requires further investigation. Our results indicate that MSC infusion is effective and safe in a mouse model of NHB liver transplantation.

## Discussion

DCDs, formerly known as “non-heart-beating donors”, exhibit notably different pathophysiology from that of brain death donors. DCDs can be divided into “controlled” and “uncontrolled” categories according to the Maastricht classification [44]. Although there has been considerably renewed interest in DCD donors to increase the pool of available organs, there are challenges associated with the additional hypoxic damage in DCD grafts, such as the increased incidence of primary non-function, early dysfunction, and cholangiopathy in controlled DCD transplantation [5–9]. Additionally, uncontrolled DCD grafts that experience warm ischaemic time of more than 30 min are excluded from transplantation worldwide, owing to the higher incidence of primary non-function, which results in a great loss of donated organs [45].

Innovative strategies and research are needed to improve the application of DCD liver grafts. However, animal studies have primarily focused on graft preservation, including ex vivo machine perfusion and modified perfusates in rat or swine models [46–50]. To our knowledge, only Liu et al. have reported syngeneic liver transplants from DCD mice; however, the surgery in their study was performed without hepatic artery reconstruction, which differs from clinical transplant procedures [51]. To strictly adhere to clinical surgery procedures, we first attempted liver transplantation from C57Bl/6 to C57Bl/6 mice; however, because of hepatic artery variations, the transplant surgery success rate was only approximately 30 %, which was not suitable for establishing an animal model. Because CBF1 mice have heterosis and do not reject liver grafts from C57Bl/6 mice, we then attempted liver transplantation from C57Bl/6 to CBF1 mice. Unexpectedly, the success rate of transplantation with hepatic artery reconstruction reached 90 %, and the survival rate and graft histology in this mouse model were consistent (Additional file 1: Figure S1). On the basis of a large amount of preliminary data, we established an optimal arterialized mouse NHB liver transplantation model characterized by 10 min of NHB time and 120 min of cold ischaemic time. The NHB_5min_ group showed no significant differences compared with the NHB_0min_ group in terms of serum ALT/AST levels, Suzuki scores, and 14-day survival rates, thus indicating that warm ischaemia for less than 5 min did not induce obvious graft injury or damage. According to the calculation of Giraud et al. [52], 5 min of warm ischaemia in mice is equivalent to 42.8 min in humans. Thus, human DCD liver grafts with a cardiac arrest time of less than 42.8 min may be acceptable, a result consistent with the clinical criterion that DCD grafts used within 30 min of warm ischaemia are safe [45]. Furthermore, mRNA levels of pro-inflammatory cytokines associated with the Th1/Th2 response, with the exception of IL2 and IL4, decreased with increased NHB duration. The exact mechanism underlying these results remains unclear, but prolonged NHB time may potentially induce severe injury or even apoptosis of intrahepatic non-parenchymal cells, ultimately resulting in decreased mRNA levels for the majority of Th1/Th2 cytokines. However, it is unknown why IL2 and IL4 are exceptions to this trend, and further investigation is required.

MSCs have powerful immunomodulatory and regenerative capacities, thus making them a promising therapeutic option for treatment of clinical diseases. However, clinical trials investigating MSCs in liver transplantation are still in process, and the ultimate effects of MSCs on prolonged graft survival, reduced transplantation side effects, and concomitant therapy have yet to be evaluated [53].

In our study, MSC infusion dramatically ameliorated NHB-induced graft injury and increased recipient survival post-transplantation. Mechanistically, MSCs inhibited Kupffer cell apoptosis by producing PGE2 in an ERK1/2-dependent manner. Together, protected Kupffer cells and infused MSCs suppressed Th1/Th17 cells, which further decreased chemokine expression and inhibited inflammatory cell infiltration, thereby resulting in a reduction in NHB-induced graft injury. Furthermore, 10 min of NHB graft injury was reversed by MSC infusion. Graft injury in the MSC infusion group was not significantly different compared with grafts in the NHB_0min_ group, thus suggesting promisingly clinical application of MSCs in uncontrolled DCD grafts. Uncontrolled DCD liver grafts with warm ischaemia time of less than 85.6 min may achieve outcomes comparable to those of controlled organs if combined with MSC infusion. Additionally, the lack of tumourigenesis observed during a prolonged observation time of 3 months indicates the safety and effectiveness of MSC infusion. However, additional clinical studies must be performed to verify the feasibility of our proposed method.

Kupffer cells, known as liver-resident macrophages, account for approximately 30 % of liver non-parenchymal cells and more than 50 % of resident macrophages throughout the entire body [54–56] and play an important role in immunomodulation, phagocytosis, biochemical attack, and liver IRI [57]. Kupffer cells create an inflammatory milieu by generating cytotoxic ROS, cytokines such as TNFα and IL1β, and neutrophil-associated chemokines such as CXCL1, CXCL2, CCL2, and CXCL10 [58–63]. In contrast, IL10 released by Kupffer cells may induce anergy in T cells and protect tissues against excessive injury [64, 65].

Interestingly, in our study, the number of F4/80-positive Kupffer cells decreased rapidly with the extension of warm ischaemia time, whereas MSC infusion restored Kupffer cells by inhibiting apoptosis. The protected Kupffer cells suppressed Th1/Th17 cells and depressed the intrahepatic infiltration of neutrophils and T cells by reducing cytokine and chemokine secretion, thus resulting in reduced graft injury. Moreover, the protective effects of MSCs on NHB liver grafts were dependent on the presence of Kupffer cells. Together, our data provide the first evidence of the extremely important role of Kupffer cells in determining NHB liver graft quality. In the future, Kupffer cells or F4/80 expression levels may be valuable in evaluating the quality of DCD liver grafts and predicting the prognosis of DCD liver transplantation.

The paracrine effects of MSCs are critical components of their immunomodulatory functions. Among numerous soluble factors, PGE2 is the most well-characterized, affecting Th17 differentiation and regulating modulatory macrophages and dendritic cells [66, 67]. In vitro data revealed the important role of PGE2 in suppressing Kupffer cell apoptosis. We also elucidated the involvement of the TLR4-ERK1/2-Fas/FasL-caspase3 signalling pathway in this process. Notably, the underlying mechanism revealed in our study broadens insight and allows for a better understanding of how MSCs protect NHB grafts (Additional file 2: Figure S2); however, other mechanisms involved in this protection require further investigation.

## Conclusions

In conclusion, we provide the first report of an optimal arterialized mouse NHB liver transplantation model for DCD-related research and reveal the profound protective effects of MSC infusion on NHB liver grafts, which dramatically improved recipient survival post-transplantation. Moreover, we also demonstrate the critical role of PGE2 in inhibiting Kupffer cell apoptosis via the TLR4-ERK1/2-caspase3 pathway. This work presents a promising and feasible option for the clinical application of MSC infusion to protect DCD liver grafts and prolong post-transplant survival in the future.

## Additional files

Additional file 1: Figure S1. C57-CBF1 transplantation showed no difference in survival and histology compared with C57-C57. (a) Photographs of NHB mice liver transplantation model. 1: Prolonged hepatic artery of graft; 2: aorta abdominalis of recipient; 3: portal vein reconstruction; 4: inferior vena cava reconstruction; 5: reconstructed hepatic artery with blood flow. (b) 14-days survival rate of C57-CBF1 showed no difference compared with C57-C57 (*N* = 10 for each group, 100 % vs. 90 %, *P* = 0.3173), which had a much higher success rate in hepatic artery reconstruction (90 % vs. 30 %). (c) Histological examination of liver grafts in each group at 6 h post-transplantation (haematoxylin and eosin staining). (d) Suzuki score of graft injury showed no difference between two groups (1.667 ± 0.2108 vs. 1.833 ± 0.1667, *N* = 6 for each group, *P* = 0.5490). (DOCX 1045 kb)
Additional file 2: Figure S2. Possible mechanisms for MSCs protecting NHB liver grafts. (a) After NHB liver transplantation, warm and cold ischemia/reperfusion injury would promote the expression of TLR4 on the surface of Kupffer cells. TLR4 could inhibit activation of ERK1/2 resulting in elevated expression of Fas/FasL and cleaved-caspase3. These changes lead to apoptosis of Kupffer cells with a large amount of pro-inflammatory cytokine and chemokine release. These cytokines and chemokines promote the inflammatory response and inflammatory cell infiltration into liver grafts. Finally, aggravated liver graft injury reduced survival rate after NHB liver transplantation. (b) MSCs could secrete PGE2, which would inhibit the elevated expression of TLR4 on Kupffer cells after NHB liver transplantation. Then activated ERK1/2 decreased expression of Fas/FasL and cleaved-caspase3, resulting in restoration of Kupffer cells. These changes suppressed Th1/Th17 cytokine and chemokine release, and reduced neutrophil and T-cell infiltration. Finally, liver grafts of good quality guaranteed a dramatic increase in the survival rate of recipients. (DOCX 272 kb)

## Abbreviations

ALT: Alanine aminotransferase
AST: Aspartate aminotransferase
CCL: C-C motif ligand
CXCL: C-X-C motif ligand
DCD: Donation after cardiac death
DMEM: Dulbecco’s modified Eagle’s medium
FBS: Foetal bovine serum
GdCl_3_: Gadolinium chloride
H_2_O_2_: Hydrogen peroxide
IDO: Indoleamine 2,3-dioxygenase
IFN: Interferon
IL: Interleukin
iNOS: Inducible nitric oxide synthase
IRI: Ischaemia/reperfusion injury
IVC: Individually ventilated cages
MPO: Myeloperoxidase
MSC: Mesenchymal stem cell
NHB: Non-heart-beating
PBS: Phosphate-buffered saline
PGE2: Prostaglandin E2
RT-PCR: Real-time polymerase chain reaction
TGF: Transforming growth factor
TNF: Tumour necrosis factor
TUNEL: Terminal deoxynucleotidyl transferase dUTP nick end labelling
