# Supplementary material for: Mesenchymal stem cells improve mouse non-heart-beating liver graft survival by inhibiting Kupffer cell apoptosis via TLR4-ERK1/2-Fas/FasL-caspase3 pathway regulation
Source: Stem Cell Res Ther. 2016 Oct 27;7:157. doi: 10.1186/s13287-016-0416-y (PMC5084468; doi:10.1186/s13287-016-0416-y)
Supplement: Additional file 1: Figure S1. — C57-CBF1 transplantation showed no difference in survival and histology compared with C57-C57. (a) Photographs of NHB mice liver transplantation model. 1: Prolonged hepatic artery of graft; 2: aorta abdominalis of recipient; 3: portal vein reconstruction; 4: inferior vena cava reconstruction; 5: reconstructed hepatic artery with blood flow. (b) 14-days survival rate of C57-CBF1 showed no difference compared with C57-C57 (N = 10 for each group, 100 % vs. 90 %, P = 0.3173), which had a much higher success rate in hepatic artery reconstruction (90 % vs. 30 %). (c) Histological examination of liver grafts in each group at 6 h post-transplantation (haematoxylin and eosin staining). (d) Suzuki score of graft injury showed no difference between two groups (1.667 ± 0.2108 vs. 1.833 ± 0.1667, N = 6 for each group, P = 0.5490). (DOCX 1045 kb) [file 13287_2016_416_MOESM1_ESM.docx]

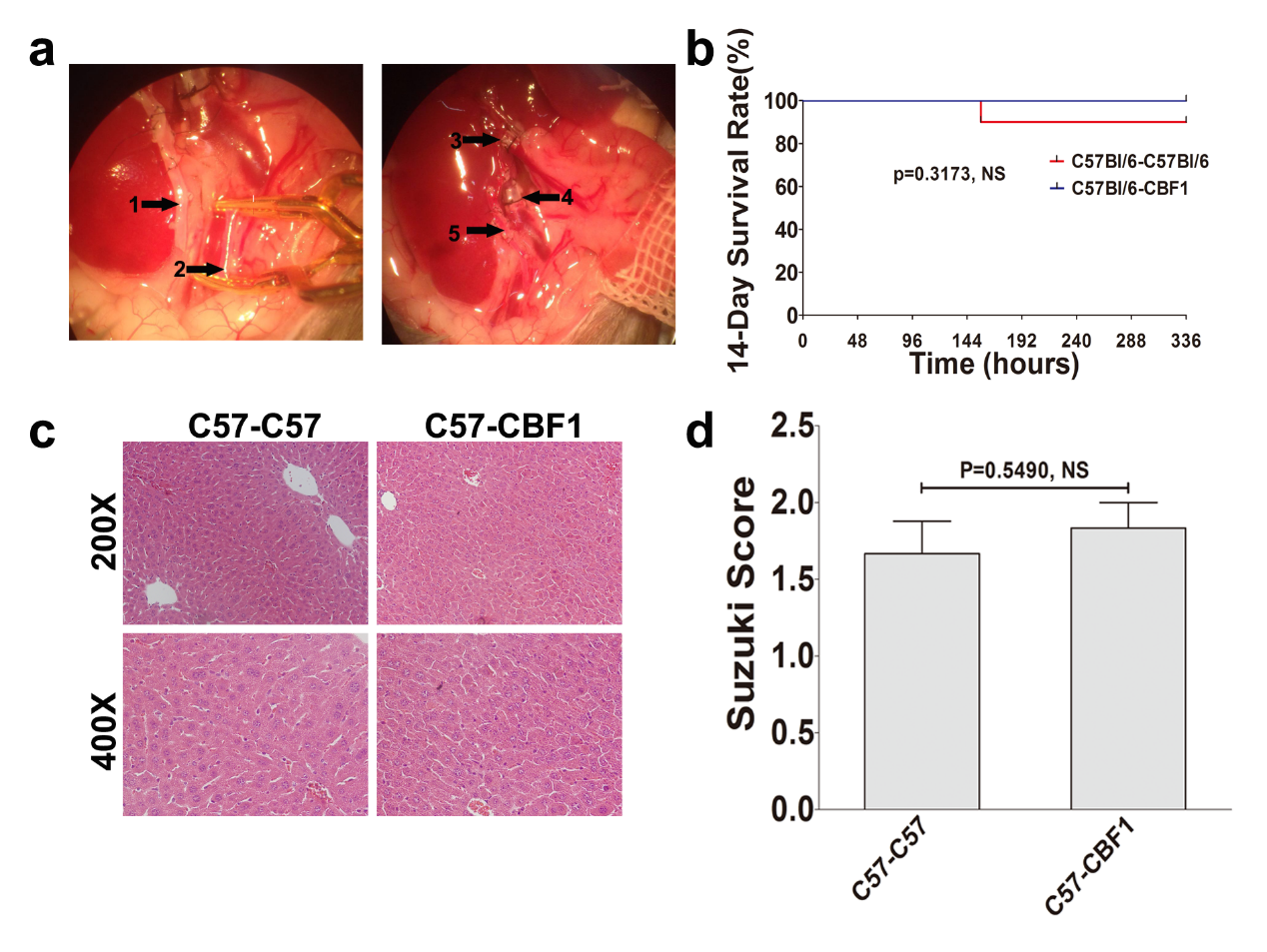


**Figure S1. C57-CBF1 transplantation showed no difference in survival and histology compared with C57-C57.**

**(a)** Photographs of NHB mice liver transplantation model. 1: prolonged hepatic artery of graft; 2: aorta abdominalis of recipient; 3: portal vein reconstruction; 4: inferior vena cava reconstruction; 5: reconstructed hepatic atery with blood flow. **(b)** 14-days survival rate of C57-CBF1 had no difference with C57-C57 (N=10 for each group, 100% vs. 90%, P=0.3173), which had a much higher successful rate in hepatic artery reconstruction (90% vs. 30%). **(c)** Histological examination of liver grafts in each group at 6h post-transplantation (H&E staining). **(d)** Suzuki’s score of graft injury showed no difference between two groups (1.667+ 0.2108 vs. 1.833 + 0.1667, N=6 for each group, P=0.5490).
