# Supplementary material for: Mesenchymal stem cells improve mouse non-heart-beating liver graft survival by inhibiting Kupffer cell apoptosis via TLR4-ERK1/2-Fas/FasL-caspase3 pathway regulation
Source: Stem Cell Res Ther. 2016 Oct 27;7:157. doi: 10.1186/s13287-016-0416-y (PMC5084468; doi:10.1186/s13287-016-0416-y)
Supplement: Additional file 2: Figure S2. — Possible mechanisms for MSCs protecting NHB liver grafts. (a) After NHB liver transplantation, warm and cold ischemia/reperfusion injury would promote the expression of TLR4 on the surface of Kupffer cells. TLR4 could inhibit activation of ERK1/2 resulting in elevated expression of Fas/FasL and cleaved-caspase3. These changes lead to apoptosis of Kupffer cells with a large amount of pro-inflammatory cytokine and chemokine release. These cytokines and chemokines promote the inflammatory response and inflammatory cell infiltration into liver grafts. Finally, aggravated liver graft injury reduced survival rate after NHB liver transplantation. (b) MSCs could secrete PGE2, which would inhibit the elevated expression of TLR4 on Kupffer cells after NHB liver transplantation. Then activated ERK1/2 decreased expression of Fas/FasL and cleaved-caspase3, resulting in restoration of Kupffer cells. These changes suppressed Th1/Th17 cytokine and chemokine release, and reduced neutrophil and T-cell infiltration. Finally, liver grafts of good quality guaranteed a dramatic increase in the survival rate of recipients. (DOCX 272 kb) [file 13287_2016_416_MOESM2_ESM.docx]

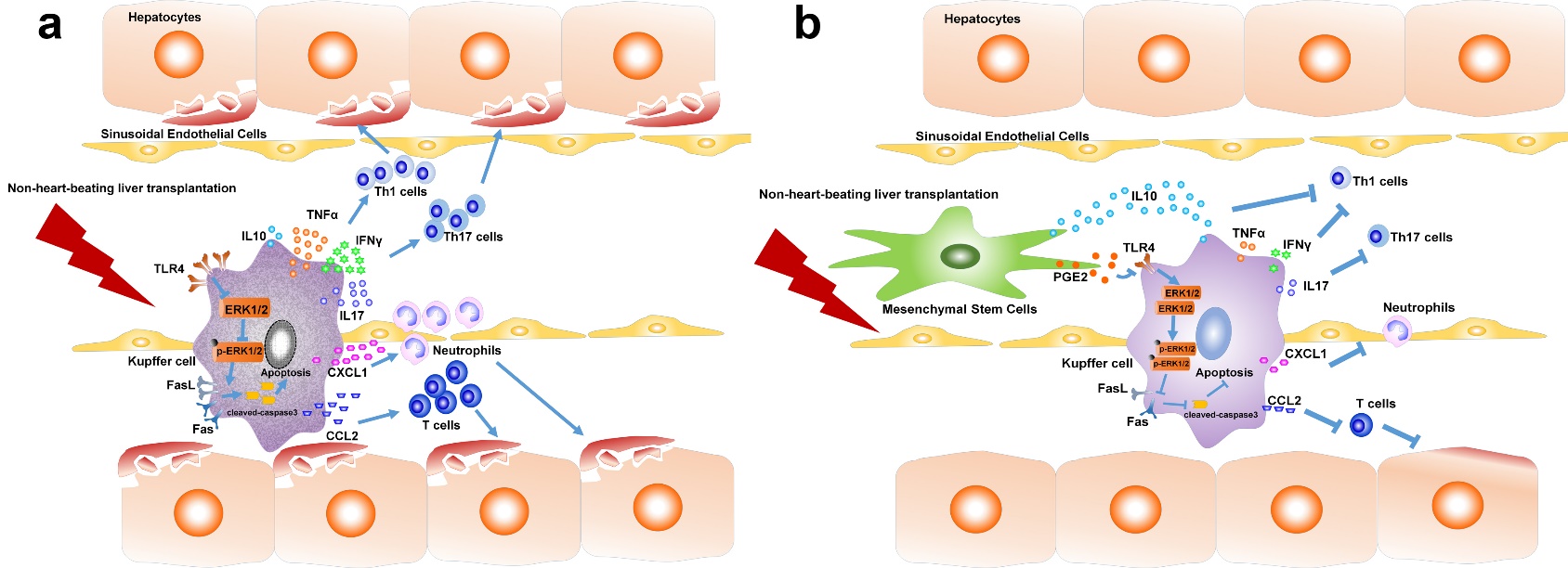


**Figure S2. Possible mechanisms of MSCs in protecting NHB liver grafts.**

**(a)** After NHB liver transplantation, warm and cold ischemia/reperfusion injury would promote the expression of TLR4 on the surface of Kupffer cells. TLR4 could inhibit activation of ERK1/2 resulting in elevated expression of Fas/FasL and cleaved-caspase3. These changes lead to apoptosis of Kupffer cells with a large amount of pro-inflammatory cytokines and chemokines release. These cytokines and chemokines promote the inflammatory response and inflammatory cells infiltration into liver grafts. Finally, aggravated liver grafts injury reduced survival rate after NHB liver transplantation. **(b)** MSCs could secret PGE2, which would inhibit the elevated expression of TLR4 on Kupffer cells after NHB liver transplantation. Then activated ERK1/2 decreased expression of Fas/FasL and cleaved-caspase3, resulting in restore of Kupffer cells. These changes suppressed Th1/Th17 cytokines and chemokines release, and reduced neutrophils and T cells infiltration. Finally liver grafts in good quality guaranteed a dramatic increase in survival rate of recipients.
